# Developing a portable natural language processing based phenotyping system

**DOI:** 10.1186/s12911-019-0786-z

**Published:** 2019-04-04

**Authors:** Himanshu Sharma, Chengsheng Mao, Yizhen Zhang, Haleh Vatani, Liang Yao, Yizhen Zhong, Luke Rasmussen, Guoqian Jiang, Jyotishman Pathak, Yuan Luo

**Affiliations:** 1 Cyberinfrastructure, University of Illinois at Chicago, Chicago, IL 60612 USA; 2 Department of Preventive Medicine, Feinberg School of Medicine, Northwestern University, Chicago, IL 60611 USA; 3 Biomedical Informatics, Mayo Clinic, Rochester, MN USA; 4 Health Informatics, Weill Cornell Medicine, New York, NY USA

## Abstract

**Background:**

This paper presents a portable phenotyping system that is capable of integrating both rule-based and statistical machine learning based approaches.

**Methods:**

Our system utilizes UMLS to extract clinically relevant features from the unstructured text and then facilitates portability across different institutions and data systems by incorporating OHDSI’s OMOP Common Data Model (CDM) to standardize necessary data elements. Our system can also store the key components of rule-based systems (e.g., regular expression matches) in the format of OMOP CDM, thus enabling the reuse, adaptation and extension of many existing rule-based clinical NLP systems. We experimented with our system on the corpus from i2b2’s Obesity Challenge as a pilot study.

**Results:**

Our system facilitates portable phenotyping of obesity and its 15 comorbidities based on the unstructured patient discharge summaries, while achieving a performance that often ranked among the top 10 of the challenge participants.

**Conclusion:**

Our system of standardization enables a consistent application of numerous rule-based and machine learning based classification techniques downstream across disparate datasets which may originate across different institutions and data systems.

## Introduction

The Electronic Health Record (EHR) is often described as “a longitudinal electronic record of patient health information generated by one or more encounters in any care delivery setting. Included in this information are patient demographics, progress notes, problems, medications, vital signs, past medical history, immunizations, laboratory data and radiology reports.” [1] As medical care becomes more data-driven and evidence-based, these EHRs become essential sources of health information necessary for decision-making in all aspects of patient assessment, phenotyping, diagnosis, and treatment.

These EHRs contain both a) structured data such as orders, medications, labs, diagnosis codes and unstructured data such as textual clinical progress notes, radiology and pathology reports. While structured data may not require significant preprocessing to derive knowledge, Natural Language Processing (NLP) techniques are commonly used to analyze unstructured data. This unstructured data can feed into a variety of secondary analysis such as clinical decision support, evidence-based practice and research, and computational phenotyping for patient cohort identification [2, 3]. Additionally, manual labeling of a large volume of unstructured data by the experts can be very time-consuming and impractical when used across multiple data sources. Automated information extraction from unstructured data through NLP provides a more efficient and sustainable alternative to the manual approach [2].

As summarized in a 2013 review by Shivade et al. [4], early computational phenotyping studies were often formulated as supervised learning problems wherein a predefined phenotype is provided, and the task is to construct a patient cohort matching the definition’s criteria. Unstructured clinical narratives may summarize patients’ medical history, diagnoses, medications, immunizations, allergies, radiology images, and laboratory test results, in the form of progress notes, discharge reports etc. and provide a valuable resource for computational phenotyping [5]. While we refer the readers to reviews such as [4, 6] for more details on phenotyping methods, we point out that information heterogeneity in clinical narratives asks for development of portable phenotyping algorithms. Boland et al. [7] highlighted the heterogeneity apparent in clinical narratives due to the variance in physicians’ expertise and behaviors, and institutional environments and setups. Studies have applied Unified Medical Language System (UMLS) or other external controlled vocabularies to recognize the various expressions of the same medical concept and standard UMLS annotations are generally considered a must for portable phenotyping [8, 9].

Our main aim was to introduce portability to the ongoing research efforts on NLP-driven phenotyping of unstructured clinical records. To this end, we leveraged a well-defined phenotyping problem, i2b2 Obesity Challenge, to perform a pilot study and introduced new steps to this multi-class and class-unbalanced classification problem for portability. We extracted structured information from 1249 patient textual discharge summaries by parsing each record through a context-aware parser (MetaMap [10]) and mapped all of the extracted features to UMLS’s Concept Unique Identifiers (CUIs). MetaMap’s output was then stored in a MySQL database using the schemas defined in the Observational Medical Outcomes Partnership (OMOP) Common Data Model (CDM), a data standardization model championed by the Observational Health Data Sciences and Informatics (OHDSI) collaborative.

We recognize the usefulness of existing rule-based (e.g., RegEx-driven) NLP systems and enable our system to introduce/improve their portability by storing key components of rule-based NLP systems as stand-off annotations [11] using the format defined in the OMOP CDM. We explore the tradeoff between phenotyping accuracy and portability, which has been largely ignored but of critical importance. We evaluated a combination of rule-based (RegEx-driven) and machine learning approaches to assess the trade-off through an iterative manner for obesity and its 15 comorbidities. We ran four types of machine learning algorithms on our dataset, and conducted multiple iterations of optimizations for a balanced trade-off between classification performance and portability. In particular, Decision Tree resulted in the best performance with the F-Micro score for intuitive classification at 0.9339 and textual classification at 0.9546 and the F-Macro score for intuitive classification at 0.6509 and textual classification at 0.7855.

## Methods

Our portable NLP system is based on sequential activities that form an NLP pipeline with six major components: a) Data Preparation and Environmental Setup, b) Section and Boundary Detection, c) Annotation Feature Extraction and Mapping, d) Regular Expression matches as Annotations, e) Classification and f) Performance Tuning.

### Environmental setup and data preparation

Data preparation, as often is the case, can be the most time-consuming part of any data analytics project and our system development journey was not an exception to the rule. Our dataset, a single file with textual discharge summaries of 1249 patients, needed data clean-up and data staging for further data reduction. In the data clean-up step, we identified multiple abbreviations that were used to explain clinical or demographical features within our master file. While these abbreviations are useful for expediting the note taking process, they need to be translated back to full terms for the context-aware MetaMap parser to properly label them as a medical concept. For this deabbreviation, we used popular deabbreviation Perl script that was created by Solt et al. [12]. The Perl script relies on Regular Expression (RegEx) pattern matching and replacement to deabbreviate terms back to long form. However, the script required us to first convert our text file into XML format. For this, we created a Python script to read each record and convert it to an XML document.

The next step was to split the master file into individual patient records. We utilized Python and RegEx to search for the end of record tags and utilized that information to formulate new files for each record. Individual patient files are required by MetaMap as it tracks the position of each concept from the start of each patient record. Our end of record keyword was ‘*[record_end]*’ that facilitated boundary detection and the downstream split into new files. A master file with 1249 patient records has been split into 1249 individual patient files.

### Section and boundary detection

Post data-preparation, our goal was to obtain a certain structure from the unstructured data. Upon visual inspection of patient documents, we observed the presence of sections within each document such as ‘PRINICIPAL DIAGNOSIS’ and ‘HISTORY OF PRESENT ILLNESS’. Based on our clinical knowledge and visual inspection of our records, we compiled a list of 15 such sections with section heading and an auto-generated unique section id. Each patient record was then parsed using string matching in Python against the compiled dictionary to detect section boundary.

For each of the 1249 patient files, we conducted string matching from the list of pre-coded sections mentioned above. Once a section heading was detected, we noted the index of the section start position (i.e. section1_start_). We continued to parse the file until we identify the starting index of a new section (i.e. section2_start_). Therefore, the section1_end_ boundary was defined as section2_start_ – 1. We retained all identified sections and their boundaries for each record temporarily in our Python code.

### Annotation feature extraction and mapping

**Fig. 1:**
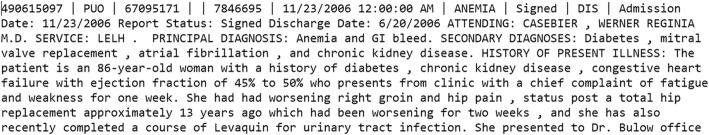
A snippet of the patient input file

**Fig. 2:**
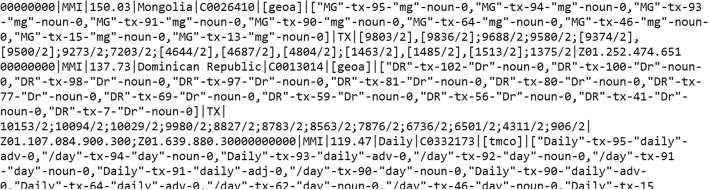
A snippet of MetaMap output record

MetaMap is an excellent tool that can map clinical text to the UMLS Metathesaurus concepts, which can be regarded in general as NLP (automated) annotations. MetaMap uses a knowledge-intensive approach based on symbolic, NLP and computational-linguistic techniques [10]. Each patient file (Fig. 1) was sequentially passed through the MetaMap parser and its output was stored in individual output files (Fig. 2). We then mapped relevant MetaMap output elements to the OMOP CDM “*Note_NLP*” Table 1.

**Table 1:** Note_NLP table data elements

Column name	Description
note_nlp_id	A unique identifier for each term extracted from a note. A randomly generated auto-incremented number.
note_id	A foreign key. The note_id from the Note table from the note the term was extracted from.
section_concept_id	The representation of the section that extracted concept belongs to.
snippet	A small window of the text that extracted concepts belong to.
offset	Provided by the MetaMap in the output file.
lexical_variant	The actual phrase text that MetaMap generates.
note_nlp_concept_id	The concepts or CUIs.
nlp_system	NLP tool.
nlp_date_time	Date and Time of creation/running

By utilizing the Common Data Model, we introduced standardization and portability in our system. Our system then sequentially parses each output file to load identified concepts (CUIs) including their offset (positional index) into the database. Then each loaded row, based on the offset, gets assigned to a specific section id. It is important to tag concepts to specific sections because based on the section, that concept may or may not be included as a feature for the classification.

### Regular expression matches as annotations

Rule-based systems, and in particular systems that use regular expressions, often prove to be highly effective in tackling medical NLP problems. For example, in the i2b2 Obesity challenge, Solt et al. [12] built a completely rule-based system that ranked first place in the intuitive task and second place in the textual task and overall first place. We value the usefulness of many existing rule-based systems and recognize the importance to introduce or improve their portability for them to be reused, adapted or extended to new corpora or phenotyping problems. This motivates us to store the key components (e.g., regular expression matches) as annotations in a common data format. For a medical record, there usually are a number of words or sentences in the record that highly suggest its category, while most of the other words or sentences are uninformative or even misleading. For example, if we capture a phrase “no evidence of coronary artery disease” from the record, it should probably be assigned as ‘Absent’ of CAD. We want to record the position of the key sentences or phrases that can help to make the classification decision.

**Fig. 3:**
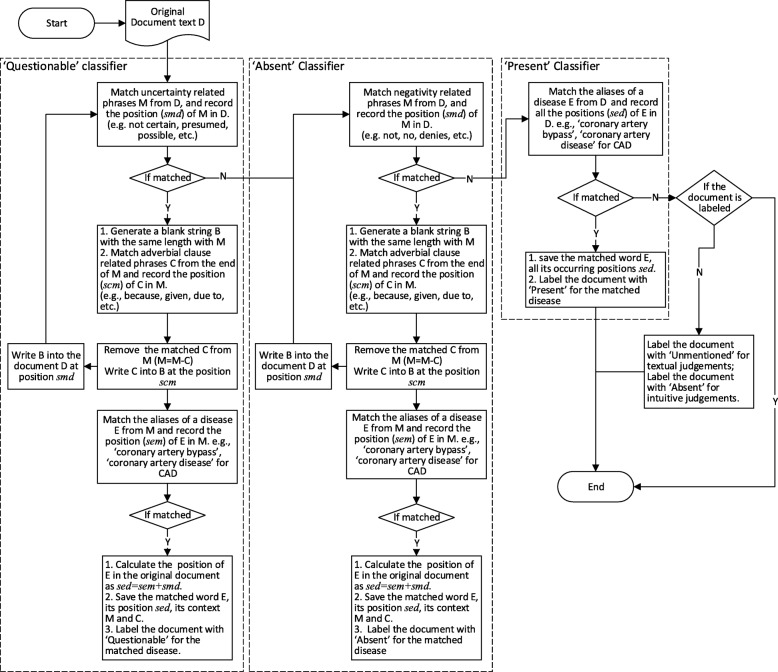
The word position recording process in our work

As Solt’s rules [12] can achieve better classification results, we follow Solt’s rule to match the category-related words or sentences. We additionally record the position of the key words or phrases when matching a RegEx, which can help to locate the key words in the original medical record. Solt’s did not record the location of the word, he just removed the matched phrase from the original document for the next step match. This would change the position of the words and will make the recording of the original position difficult. For example, the Q-classifier-based rules remove the uncertainty phrases from a document before the document goes to the N-classifier for ‘Absent’ classification. Thus, when we record the position of an ‘Absent’-related word, it is no longer the position in the original record. To overcome the difficulty of recording word positions in the original document, instead of removing the matched RegEx, we replace the matched RegEx with a blank string of the same length to keep document length unchanged. Then, successive RegEx match can record the position of a word in the original text. Our word position recording process together with the document annotation process is outlined in Fig. 3. Figure 3 recaps the rule-based classification in Solt’s paper [12], and further adds our regular expression match location algorithms in order to persist the RegEx matches to OMOP CDM tables. Our design can take as input any text span. For any text span passed to the system, our algorithm will return the regular expression match position in this text span.

For each document, there can be 3 tables to save the key phrases corresponding to ‘Questionable’, ‘Absent’ and ‘Present’. For each of the tables, there are 3 fields described as follows.

- *disease*: the name of the disease.
- *dis_alias*: the matched alias name of the disease.
- *dis_pos*: the matched position of this match in the original document (start and end position by character offset).

For ‘Questionable’ and ‘Absent’ categories, the context of the matched disease alias is also very important. The matched RegEx should be in a sentence related to uncertainty or negation respectively. Thus, we add two more fields in the tables for words related ‘Questionable’ and ‘Absent’ to save the context of the matched RegEx. The two fields are described as follows.

- *sentence*: the sentence or phrase containing this match.
- *sen_pos*: the position of this sentence or phrase in the original document (start and end position by character offset).

**Fig. 4:**
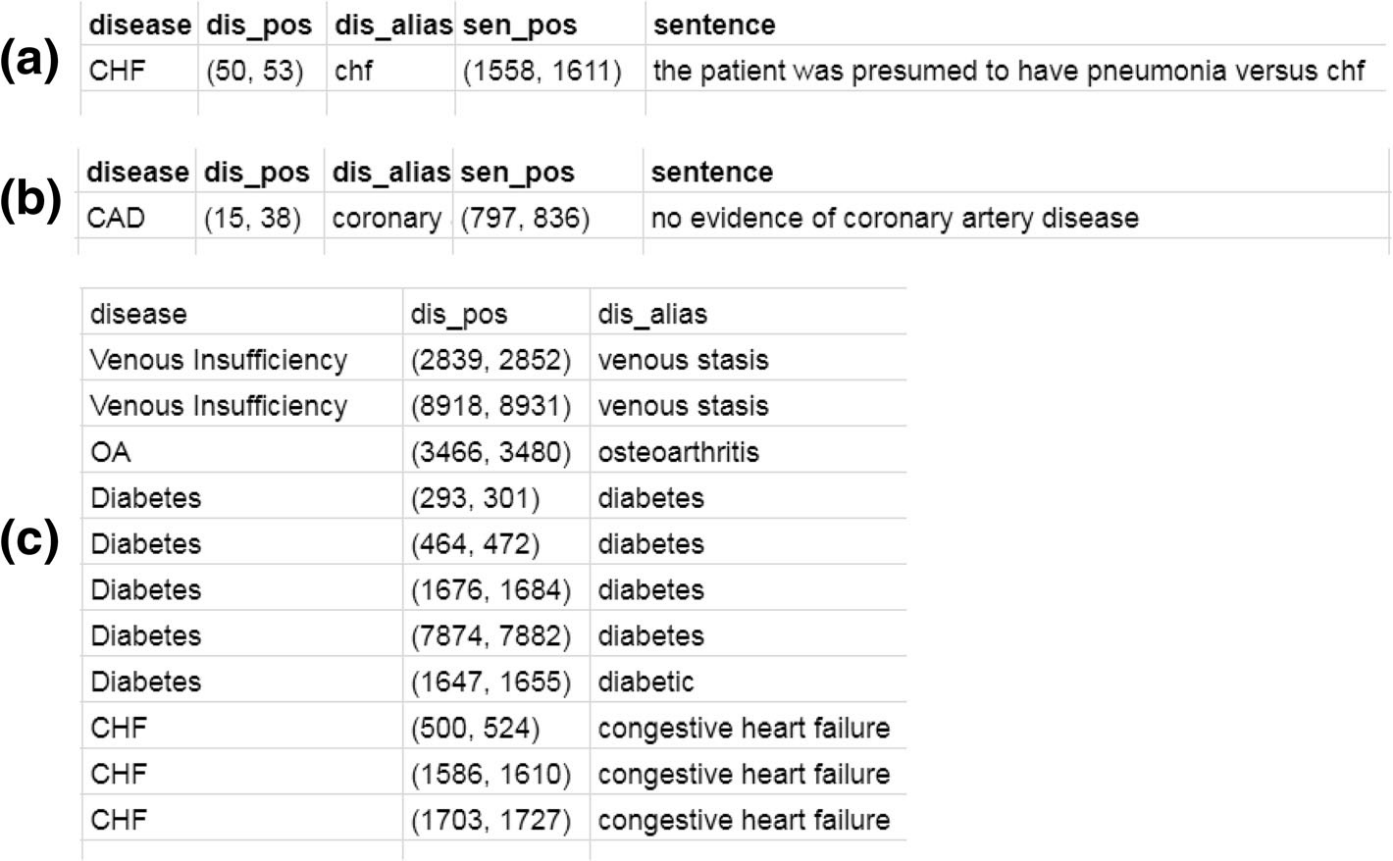
A sample of the matched regex tables. **a** the table for words related to ‘Questionable’; (**b**) the table for words related to ‘Absent’; (**c**) the table for words related to ‘Present’

Figure 4 shows a sample of the three tables. From these three tables, we can easily populate the OMOP CDM’s “*NOTE_NLP*” Table 1. For example, columns offset (in the whole record) and snippet are readily computed from *dis_pos* and *sen_pos*. The column *lexical_variant* can be populated with *dis_alias*.

### Classification

Since rule-based (RegEx-driven) approaches are regarded less portable between different EHR systems, we develop a machine learning based approach to improve the portability, and evaluated a range of rule-based approaches, machine learning algorithms and their mixtures to assess the trade-off between phenotyping accuracy and portability.

For each patient record, we obtain all the CUIs from the MetaMap parser. We then count the number of each CUI. This will represent the frequency of occurrence of the CUI in a medical record and serves as a feature of the record. Thus, we can construct the feature matrix based on the records and their corresponding CUIs’ frequency. We train a classification model on this feature matrix and the labels corresponding to training records and then evaluate the model using the feature matrix corresponding to the test records. In our experiment tasks, the class labels are ‘Present’, ‘Absent’ and ‘Questionable’ for intuitive judgments, and ‘Present’, ‘Absent’, ‘Questionable’ and ‘Unmentioned’ for textual judgments. To systematically evaluate the trade-off between model accuracy and portability on these data, we implement four classification methods for the classification tasks, i.e., logistic regression (LR) [13], support vector machine (SVM) [14], decision tree (DT) [15, 16] and random forest (RF) [17].

### Performance tuning

For the classifiers, there are some parameters to be tuned to get better classification results. In our experiments, the parameters of the classifiers are tuned by the 3-fold cross-validated grid-search over a parameter grid [18, 19]. For the 4 classifiers we implemented, their parameter grids are defined in Table 2. For each classifier, we performed the classification for six iterations to find a better configuration for classification: a) with all CUIs, b) eliminate features from unnecessary sections, c) restrict features from clinically relevant semantic types; restrict classification to classes with statistically significant samples and then again run d) classification with all CUIs, e) eliminate features from unnecessary sections, and f) restrict features from clinically relevant semantic types.

**Table 2:** The parameter grids for grid search

Classifier	Parameter grid
LR	‘C’:[0.01,0.1,1,10,100]
SVM	‘C’:[0.01,0.1,1,10,100], ‘kernel’:[‘linear’, ‘rbf’]
DT	‘criterion’:[‘gini’,'entropy’]
RF	‘n_estimators’:[5,10,30,50,80,100], ‘criterion’:[‘gini’,'entropy’]

## Results and discussion

In our experiments, the classification performances were evaluated using micro- and macro-averaged precision (P), recall (R), and F-measure (F) [20]. Because the machine learning methods may not very effective for small sample classifications, we conducted two experiments for classification for all classes and only for the major (more populated) classes, respectively, and compared their results. In the case of classification for all classes, this setting uses standard UMLS CUI features to classify all classes for all disease phenotypes, and is considered most portable. On the contrary, entirely using Solt’s rule-based system is considered the least portable as it contains the most amount of customization (and certainly it produces the top results among challenge participants). In the middle of the spectrum, there is the case of classification only for the major classes, as it integrates rule-based features using a minimal principle (where there is simply not enough training data) while retaining the standard annotation features as much as possible. Much of our results and discussions should be interpreted in the context of exposing the trade-off between portability and accuracy, as well as the parameter optimization when taking the middle-ground approach of combining rule-based features and standard UMLS CUI features.

### Classification for all classes

Based on the above settings we obtain the classification results for all CUIs in Table 3 (We only list the overall classification results here). From Table 3, we find that decision tree can achieve the best classification results among these classifiers.

**Table 3:** The classification results on all CUIs corresponding to the original records

	P-Micro	P-Macro	R-Micro	R-Macro	F-Micro	F-Macro
Intuitive						
LR	0.8719	0.5792	0.8719	0.5509	0.8719	0.5618
SVM	0.8727	0.5776	0.8727	0.5537	0.8727	0.5632
DT	**0.9281**	**0.6113**	**0.9281**	**0.6116**	**0.9281**	**0.6115**
RF	0.8524	0.5626	0.8524	0.5349	0.8524	0.5454
Textual						
LR	0.8846	0.4379	0.8846	0.4195	0.8846	0.4268
SVM	0.8886	0.4384	0.8886	0.4243	0.8886	0.4300
DT	**0.9436**	**0.5127**	**0.9436**	**0.5115**	**0.9436**	**0.5121**
RF	0.8621	0.4220	0.8621	0.4044	0.8621	0.4112

For each task, the best results are bolded

To disclose how a section (e.g. Family History) in the records can affect the classification results, we filter out the family history related CUIs and perform the classifications. The results are listed in Table 4. Comparing Tables 3 and 4, all the classifiers except LR can achieve higher performances without the family history than performances with it, which may indicate that family history may mislead the classification when only considering the record text for classification.

**Table 4:** The classification results without family history related CUIs

	P-Micro	P-Macro	R-Micro	R-Macro	F-Micro	F-Macro
Intuitive						
LR	0.8716	0.5794	0.8716	0.5503	0.8716	0.5615
SVM	0.8735	0.5780	0.8735	0.5546	0.8735	0.5640
DT	**0.9331**	**0.6159**	**0.9331**	**0.6149**	**0.9331**	**0.6154**
RF	0.8627	0.5685	0.8627	0.5462	0.8627	0.5551
Textual						
LR	0.8836	0.4372	0.8836	0.4189	0.8836	0.4262
SVM	0.8895	0.4391	0.8895	0.4248	0.8895	0.4306
DT	**0.9475**	**0.5284**	**0.9475**	**0.5199**	**0.9475**	**0.5238**
RF	0.8618	0.4210	0.8618	0.4049	0.8618	0.4112

For each task, the best results are bolded

We also conduct experiments on a list of selected CUIs without family history. We restrict our features in 15 types of CUIs which are considered most related to clinical tasks, based on clinical experiences [21] (Table 5). The classification results are shown in Table 6. Comparing Tables 4 and 6, except for DT which can achieve the highest performances among the 4 classifiers, all other classifiers can achieve better classification performances than the performances with all CUIs. This may indicate that the 15 clinically relevant semantic types of CUIs are quite informative for classification.

**Table 5:** Fifteen semantic types selected for clinical feature representations [21]

CUI	Semantic group	Semantic type description
T017	Anatomy	Anatomical Structure
T022	Anatomy	Body System
T023	Anatomy	Body Part, Organ, or Organ Component
T033	Disorders	Finding
T034	Phenomena	Laboratory or Test Result
T047	Disorders	Disease or Syndrome
T048	Disorders	Mental or Behavioral Dysfunction
T049	Disorders	Cell or Molecular Dysfunction
T059	Procedures	Laboratory Procedure
T060	Procedures	Diagnostic Procedure
T061	Procedures	Therapeutic or Preventive Procedure
T121	Chemicals & Drugs	Pharmacologic Substance
T122	Chemicals & Drugs	Biomedical or Dental Material
T123	Chemicals & Drugs	Biologically Active Substance
T184	Disorders	Sign or Symptom

**Table 6:** The classification results without family history on 15 types of selected CUIs

	P-Micro	P-Macro	R-Micro	R-Macro	F-Micro	F-Macro
Intuitive						
LR	0.9024	0.6040	0.9024	0.5763	0.9024	0.5874
SVM	0.9077	0.6055	0.9077	0.5831	0.9077	0.5924
DT	**0.9299**	**0.6131**	**0.9299**	**0.6129**	**0.9299**	**0.6130**
RF	0.8784	0.5849	0.8784	0.5559	0.8784	0.5671
Textual						
LR	0.9145	0.4560	0.9145	0.4410	0.9145	0.4472
SVM	0.9227	**0.5832**	0.9227	0.4532	0.9227	0.4607
DT	**0.9452**	0.4878	**0.9452**	**0.4785**	**0.9452**	**0.4807**
RF	0.8830	0.4353	0.8830	0.4195	0.8830	0.4258

For each task, the best results are bolded

### Classification for major classes

Though machine learning based approaches are portable, compared with the total rule-based classification results listed in Table 7, total machine learning based classification cannot achieve good performance. Hence, we may combine rule-based approaches and machine learning algorithms to balance the classification performance and portability.

**Table 7:** The best rule-based classification results reported in [20]

	P-Micro	P-Macro	R-Micro	R-Macro	F-Micro	F-Macro
Intuitive	0.9590	0.7485	0.9590	0.6571	0.9590	0.6745
Textual	0.9756	0.8318	0.9756	0.7776	0.9756	0.8000

Due to the limitation of machine learning methods on small samples, in this section, we perform the classification only on the major classes that have enough samples to train a machine learning model. The class labels of the minor classes that have only a few samples are generated following Solt’s rule-based method [12]. For intuitive judgments, we only use the ‘Present’ and ‘Absent’ records in the training data to train the classification model. For textual judgments, we only consider the ‘Present’ and ‘Unmentioned’ records. The classification results for major classes can be found in Tables 8, 9 and 10 corresponding to results for all the original CUIs, all the CUIs without family history and the selected 15 types of CUIs without the family history. In Tables 8, 9 and 10, the best results are bolded, and the underlined results can achieve the top 10 results reported in [20].

**Table 8:** The classification results for major classes on all CUIs corresponding to the original records

	P-Micro	P-Macro	R-Micro	R-Macro	F-Micro	F-Macro
Intuitive						
LR	0.8709	0.6457	0.8709	0.5733	0.8709	0.5960
SVM	0.8724	0.6444	0.8724	0.5770	0.8724	0.5981
DT	**0.9311**	**0.6804**	**0.9311**	**0.6374**	**0.9311**	**0.6488**
RF	0.8466	0.6226	0.8466	0.5559	0.8466	0.5765
Textual						
LR	0.8882	0.7846	0.8882	0.7085	0.8882	0.7397
SVM	0.8930	0.7858	0.8930	0.7135	0.8930	0.7434
DT	**0.9545**	**0.8167**	**0.9545**	**0.7636**	**0.9545**	**0.7854**
RF	0.8882	0.7846	0.8882	0.7085	0.8882	0.7397

For each task, the best results are bolded. The underlined results can achieve the top 10 results reported in [20]

**Table 9:** The classification results for major classes without family history related CUIs

	P-Micro	P-Macro	R-Micro	R-Macro	F-Micro	F-Macro
Intuitive						
LR	0.8723	0.6473	0.8723	0.5741	0.8723	0.5970
SVM	0.8732	0.6448	0.8732	0.5780	0.8732	0.5989
DT	**0.9339**	**0.6829**	**0.9339**	**0.6392**	**0.9339**	**0.6509**
RF	0.8559	0.6317	0.8559	0.5623	0.8559	0.5838
Textual						
LR	0.8886	0.7854	0.8886	0.7083	0.8886	0.7398
SVM	0.8938	0.7865	0.8938	0.7139	0.8938	0.7439
DT	**0.9546**	**0.8164**	**0.9546**	**0.7640**	**0.9546**	**0.7855**
RF	0.8640	0.7665	0.8640	0.6934	0.8640	0.7233

For each task, the best results are bolded. The underlined results can achieve the top 10 results reported in [20]

**Table 10:** The classification results for major classes without family history on the 15 types of selected CUIs

	P-Micro	P-Macro	R-Micro	R-Macro	F-Micro	F-Macro
Intuitive						
LR	0.9001	0.6695	0.9001	0.5979	0.9001	0.6206
SVM	0.9074	0.6725	0.9074	0.6065	0.9074	0.6274
DT	**0.9285**	**0.6783**	**0.9285**	**0.6355**	**0.9285**	**0.6467**
RF	0.8690	0.6417	0.8690	0.5740	0.8690	0.5952
Textual						
LR	0.9188	0.8037	0.9188	0.7303	0.9188	0.7608
SVM	0.9273	0.8060	0.9273	0.7388	0.9273	0.7669
DT	**0.9538**	**0.8160**	**0.9538**	**0.7633**	**0.9538**	**0.7849**
RF	0.8864	0.7823	0.8864	0.7081	0.8864	0.7386

For each task, the best results are bolded. The underlined results can achieve the top 10 results reported in [20]

From Tables 8, 9 and 10, we can draw a consistent conclusion with previous analysis that the Family History section may mislead the classification and the 15 clinically relevant semantic types of CUIs can be useful for these classifiers except DT. In addition, by combining the rule-based approach and machine learning based approaches, we can achieve a comparable classification performance with the total rule-based approach, and more importantly, this method can be portable between different EHR systems. This is as expected due to the limitation of machine learning methods on small samples. Thus, in our portable phenotyping system, we can use the rule-based method for the minor class classification and use machine learning methods for the major class classification. In the future, we plan to explore whether a richer CDM may help improve the computational phenotyping performance [22].

## Conclusion

Recently, increasing amount of patient data is becoming electronically available. To handle the explosion of EHR data, healthcare professionals and researchers will increasingly rely on automated or semi-automated computational techniques to derive knowledge from these data. Significant effort has been devoted to the implementation of open-sourced, standard-based systems to improve the portability of electronic health record (EHR)-based phenotype definitions (e.g., eMERGE [23] and PhEMA [24]). We developed a portable phenotyping system that is capable of integrating both rule-based and statistical machine learning based phenotyping approaches. Our system can mine and store both standard UMLS features and the key features of rule-based systems (e.g., regular expression matches) from the unstructured text as NLP annotations using the format defined by the OMOP CDM, in order to standardize necessary data elements. Comparing to file system based pipelines such as UIMA CAS stacks and BioC, the OMOP CDM uses a database as the persistent storage and has the advantages offered by database management systems. This includes well-defined schemas, remote queries and query optimizations. We demonstrated that we can store NLP annotations including those from concepts from standard pipelines (e.g., MetaMap), regular expression matches, and section annotations in CDM tables, which can later be used for computational phenotyping. Our system can thus enable the development of new standard UMLS feature-based NLP systems as well as the reuse, adaptation and extension of many existing rule-based clinical NLP systems. Given the highly variable nature of unstructured biomedical data and evolving machine learning techniques, future researchers may also benefit from adopting a similar iterative approach to optimizing their classification and by using mixed classification methods. However, variation in data models and coding systems used at different institutions make it difficult to conduct a large-scale analysis of observational healthcare databases. Our system is a first step to address that problem and enhances its portability by utilizing the OMOP CDM and its standardized terminologies. Once data (raw input and processed output) from multiple sources get harmonized into the Common Data Model, researchers can conduct systematic analysis at a larger scale to perfect these new secondary research techniques in biomedical data mining, Natural Language Processing, Machine Learning etc. By breaking down the barriers of institutional variability with portable systems and standardized terminologies, we can unlock the hidden potential in our biomedical and health data. We note that we have not explored how the CDM can be applied to tasks other than phenotyping/classification tasks and will leave it as future work to explore how CDM can lend value to other types of tasks as well.
